# Changes in prevalence and the cascade of care for type 2 diabetes over ten years (2005-2015): results of two nationally representative surveys in Mozambique

**DOI:** 10.1186/s12889-022-14595-7

**Published:** 2022-11-25

**Authors:** Tavares Madede, Albertino Damasceno, Nuno Lunet, Orvalho Augusto, Carla Silva-Matos, David Beran, Naomi Levitt

**Affiliations:** 1 Departament of Community Health, Faculty of Medicine, University Eduardo Mondlane, 702 Salvador Allende Ave, PO Box 257, Maputo, Mozambique; 2 Chronic Disease Initiative for Africa, Department of Medicine, Faculty of Health Sciences, University of Cape Town, Cape Town, South Africa; 3 Faculty of Medicine, Eduardo Mondlane University, Maputo, Mozambique; 4 Research Unit, Department of Medicine, Maputo Central Hospital, Maputo, Mozambique; 5 Departamento de Ciências da Saúde Pública e Forenses e Educação Médica, Faculdade de Medicina da Universidade do Porto, Porto, Portugal; 6 Laboratório para a Investigação Integrativa e Translacional em Saúde Populacional (ITR), Porto, Portugal; 7 EPIUnit - Instituto de Saúde Pública, Universidade do Porto, Porto, Portugal; 8 Ministry of Health, Maputo, Mozambique; 9 Division of Tropical and Humanitarian Medicine, University of Geneva and Geneva University Hospitals, Geneva, Switzerland

**Keywords:** NCD, Type 2 diabetes, Prevalence, Trend analysis, Mozambique, Sub-Saharan Africa, Adult population, National survey

## Abstract

**Background:**

Sub-Saharan Africa is predicted to have the steepest increase in the prevalence of diabetes in the next 25 years. The latest Mozambican population-based STEPS survey (STEPS 2005) estimated a 2.9% prevalence of diabetes in the adult population aged 25-64 years. We aimed to assess the change in prevalence, awareness, and management of diabetes in the national STEPS survey from 2014/2015 compared to 2005.

**Methods:**

We conducted an observational, quantitative, cross-sectional study following the WHO STEPS surveillance methodology in urban and rural settings, targeting the adult population of Mozambique in 2015. We collected sociodemographic data, anthropometric, and 12 hour fasting glucose blood samples in a sample of 1321 adults. The analysis consisted of descriptive measures of the prevalence of impaired fasting glucose (IFG), diabetes and related risk factors by age group, sex, and urban/rural residence and compared the findings to those of the 2005 survey results.

**Results:**

The prevalence of IFG and diabetes was 4.8% (95CI: 3.6-6.3) and 7.4% (95CI: 5.5-10.0), respectively. These prevalence of IFG and diabetes did not differ significantly between women and men. The prevalence of diabetes in participants classified with overweight/obesity [10.6% (95CI: 7.5-14.6)] and with central obesity (waist hip ratio) [11.0% (95CI: 7.4-16.1)] was almost double the prevalence of their leaner counterparts, [6.3% (95CI, 4.0-9.9)] and [5.2% (95CI: 3.2-8.6)], respectively. Diabetes prevalence increased with age. There were 50% more people with diabetes in urban areas than in rural. Only 10% of people with diabetes were aware of their disease, and only 44% of those taking oral glucose-lowering drugs. The prevalence of IFG over time [2.0% (95CI: 1.1-3.5) vs 4.8% (95CI: 3.6-6.3)] and diabetes [2.9% (95CI: 2.0-4.2) vs 7.4% (95CI: 5.5-10.0)] were more than twofold higher in 2014/2015 than in 2005. However, awareness of disease and being on medication decreased by 3% and by 50%, respectively. Though this was not statistically significant.

**Conclusions:**

While the prevalence of diabetes in Mozambique has increased from 2005 to 2015, awareness and medication use have declined considerably. There is an urgent need to improve the capacity of primary health care and communities to detect, manage and prevent the occurrence of NCDs and their risk factors.

**Supplementary Information:**

The online version contains supplementary material available at 10.1186/s12889-022-14595-7.

## Introduction

Non-communicable diseases (NCDs) account for about 71% of all deaths recorded globally every year [1]. Of the approximately 41 million deaths attributed to these diseases, 77% occur in low- and middle-income countries (LMIC) [1]. Current statistics indicate that 537 million people aged 20 to 79 years live with diabetes worldwide, of which 81% are in LMIC [2].

Factors such as rapid urbanization, an ageing population, smoking, harmful alcohol consumption, physical inactivity, and the adoption of unhealthy diets have been implicated in the increasing burden of NCDs in LMIC, affecting the most disadvantaged subgroups [3, 4]. The health systems of these countries have been characterized by scarce physical and human resources, weakened information systems, and inadequate infrastructure [5]. Current insights into the true burden of NCDs; their associated co-morbidities, disease patterns and phenotypes; as well as the main risk factors involved in their occurrence remains inadequately described in sub-Saharan Africa (SSA) [4–7].

Mozambique’s official data sources indicate that risk factors for NCDs have been increasing and mainly affect the younger population and are, therefore, implicated in early disability and mortality from NCD in the country [8]. Further, Mozambique’s first chronic disease risk surveillance survey (STEPS 2005) reported a prevalence of type 2 diabetes of 2.9% among the population aged 25 to 64 years, with a higher prevalence found in urban areas compared to rural, and an increasing trend in the prevalence with increasing age. The same study [9] reported that only 13% of people with diabetes knew about their condition, and just over a tenth took medications to lower their blood sugar levels. Other studies from SSA conducted between 2008 and 2017 have reported a prevalence of diabetes that varied from 1.4 to 9.1%, depending on the study setting, level of urbanization, age, and levels of obesity of the population studied [10–13].

This report describes the changes in type 2 diabetes prevalence and the cascade of care over a ten year period (2005-2015). As part of a community-based epidemiological survey for chronic diseases risk factors surveillance in Mozambique in 2015, we conducted a cross-sectional study to assess the prevalence of diabetes, impaired fasting glucose (IFG) and related risk factors, and examine the cascade of care for diabetes in the adult Mozambican population.

## Methods

### Study Design

We conducted a cross-sectional study following the World Health Organization (WHO) STEPS surveillance methodology in urban and rural settings, targeting the adult population of Mozambique in 2015.

### Study Setting and Participants

This study was conducted in Mozambique, located on the Eastern Coast of Africa, with a mainly subsistence agricultural-based economy and one of the lowest reported gross domestic products globally [14]. A nationally representative sample of 2181 adults, aged 25 and 64 years, was assembled from each of Mozambique’s 11 provinces through a multistage random sampling design. Sampling strategy and sample size aimed to be the same as in the previous Mozambique STEPS survey as described elsewere [15, 16]. In brief, the sample was designed based on the 2007 population census and was powered to the national and urban-rural area levels. The homeless and people living in collective residential institutions (e.g. hotels, hospitals, military facilities), were not eligible. Sampling weights were computed by the National Institute of Statistics (INE).

Participants were selected through a complex sampling design in three stages. The first stage included the selection of 120 primary sampling units (PSU, geographical units including 400 to 600 households in the urban areas and 400 to 500 households in the rural areas), with probability proportional to the number of households, stratified according to province and urban-rural areas. The second stage included the random selection of one enumeration area (geographical unit including 100 to 150 households in the urban areas and 80 to 100 households in the rural areas) within each PSU, corresponding to a total of 120 clusters. The third stage comprised an update of the list of households in each enumeration area selected, followed by random and systematic selection of 24 households. Within each selected household, all dwellers aged 15 to 64 years were listed and a maximum of two were selected, one aged 15 to 44 years and one aged 45-64 years, whenever available. When there was more than one household member in each of these age-groups, only one per group was randomly selected, using a Kish selection grid. A total of 3277 subjects were invited and 3119 agreed to participate (participation rate: 95.2%) (Supplementary Fig. 1).

### Study Procedures

The composition, organization and preparation of data collection teams are described elsewere [15]. Data collection was conducted following the three-stage requirements of the WHO STEPS Procedures Manual [17]. First, a trained fieldworker administered the questionnaire which was adapted to the Mozambican context. Second, a different fieldworker, trained to perform vital sign measurements, measured blood pressure and anthropometrics (i.e. height, weight, waist and hip circumferences). Lastly, a 12-hour finger prick fasting blood glucose (FBG) sample was collected to immediately assess glucose, total cholesterol, and HDL cholesterol concentrations. A dry method in a digital unit (CardioChek PLUS v1.05, Silver; Smart Bundle Lipid 45’s + eGlu 50’s) was used for the biochemical tests.

According to WHO standards for whole blood finger prick testing, participants were classified as 1) normoglycaemic if they had a FBG < 5.6 mmol/l; 2) having impaired fasting glucose (IFG) if their FBG ≥ 5.6 mmol/l and < 6.1 mmol/l; and 3) as diabetic if their FBG ≥ 6.1 mmol/l or if they were being treated with insulin and/or oral glucose lowering drugs [18].

Detailed sociodemographic information, measurements and laboratory results were recorded using paper-based data collection tools and then computerized centrally at the National Institute of Statistics (*Instituto Nacional de Estatística*, INE) in Maputo, between February and April 2016. The team used an interactive software for microcomputers Census and Survey Processing System (CSPRO) for data entry. We employed double data entry to allow an interactive verification process of the variable ranges, detection of inconsistencies, and quality control of the internal data flow.

### Data Analysis

Data analysis was restricted to those participants who observed the 12-hour fasting requirement and provided data for the questionnaire and physical measurements. Descriptive measures (means, frequencies, interquartile ranges, and standard deviations) were used to summarize the characteristics of the study population (sex, place of residence, age group, and education) and describe the following: prevalence of IFG; diabetes; overweight and obesity [body mass index (BMI) 25-29.9 and ≥ 30 kg/m^2^] as defined by the WHO [19]; and abdominal obesity [waist-to-height (WHtR) ratio (< 0.5, 0.5 - 0.6, ≥ 0.6) [20] and waist-to-hip (WHtH) ratio (< 0.9, ≥ 0.9)] [21]. Survey weighting estimators were employed through the “*svy”* command in Stata 15 (StataCorp, College Station, TX, USA) to account for the sample’s complex design (stratification, clustering by PSU, and unequal probability of selection of the household) and to obtain Mozambique’s national adult population representativeness. We estimated variances through the first-order Taylor series linearization and reported 95% confidence intervals (95CI). Given that a substantial fraction (39.4%) of the sample did not fulfil the fasting requirement, the imputation of FBG was performed using a multiple chain equation (MICE). Miri et al. recommended that MICE was conducted in the Box-Cox transformed continuous variables FBG, BMI, height, hip length and waist lengths [22]. The age, sex, education and strata were used to improve the imputation [22]. We generated 30 imputed datasets, assessed convergence by examining the plots, estimated the prevalence (of diabetes and IFG) in each dataset respecting the survey weights, strata and clustering, and pooled the 30 estimates into one using Rubbin rules.

We computed prevalence differences (PD) to compare the prevalence estimates of the surveys from 2014/2015 and 2005 and confidence intervals were computed according to Lachin [23]. In addition to the unadjusted analysis, we performed directed age-sex standardization of the prevalence using the 2017 census as our reference population. Furthermore, log-binomial regressions were calculated to estimate the prevalence ratio between groups. Finally, to compute the total population in each step of the cascade of care, we used Mozambique’s projected national population from 2015.

## Results

### Response rate and study participants

Data were collected from a sample of 2181 subjects aged 25 to 64 years of age. Of these, a total of 1321 (60.6%) subjects had complete data including 12-hour fasting blood samples, a completed questionnaire, and physical measurements, and were included in the analysis. Using survey frame data, we explored age, area of residence, and waist circumference biases in response rates. The excluded subjects (39.4%), who did not comply with the 12-hour fasting, were younger (mean age 29.5 years [SD 13.1] vs. 41.2 [SD 11.4]); lived mostly in urban areas (52% vs. 41%); and were leaner (female mean waist circumference 77 cm (SD 11.8) vs. 80.4 cm (SD 12.6); male mean waist circumference 74.9 cm (SD 9.4) vs. 78.5 cm (SD 10.5). A sensitivity analysis using the imputation of fasting blood glycemia (FBG) through MICE did not show significant differences in the results of diabetes and IFG point estimates and confidence intervals compared to a complete case analysis (Supplementary Tables S1 and S2).

Of subjects with complete data, the majority were women (60%), lived mainly in rural settings (68%), and were between the ages of 25 and 54 years (83%). Most had a low level of education, with < 20% having pursued more than a primary education (5th grade) **(**Table 1**)**. Age, rural-urban distribution, and educational level, reported in our survey, were similar to that which has been reported in official national numbers, however the female to male sex difference was wider (20% vs 4%) in our population.

**Table 1:** Demographic and anthropometric characteristics of the participants

	N	Unweighted distributions	Weighted distribution	
		%	%	95CI
Gender				
Female	814	61.6	**60.3**	57.5 - 63.1
Male	507	38.4	39.7	36.9 - 42.6
Place of residence				
Urban	545	41.3	31.7	23.4 - 41.2
Rural	776	58.7	**68.4**	58.8 - 76.6
Age (years)				
25 - 34	478	36.2	34.0	30.7 - 37.5
35 - 44	337	25.5	28.1	24.6 - 31.8
45 - 54	288	21.8	20.9	18.6 - 23.3
55 - 64	218	16.5	17.0	14.4 - 20.2
Mean age (SE)	1321	41.2 (0.31)	41.4 (0.44)	40.6 - 42.3
Median age (IQR)	1321	39 (31 - 50)	40 (32 - 50)	39.5 - 40.5*
Education (years)				
None	427	32.5	**33.9**	29.7 - 38.2
1 - 5	617	46.9	**49.3**	45.5 - 53.2
6 or more	272	20.7	16.8	13.5 - 20.7
Body mass index (kg/m2) - Mean (SE)	1272	23.8 (0.15)	23.2 (0.24)	22.8 - 23.7
Waist circumference, women (cm) - Mean (SE)	772	80.4 (0.45)	78.9 (0.53)	77.9 - 80.0
Waist circumference, men (cm) - Mean (SE)	504	78.5 (0.47)	78.0 (0.58)	76.9 - 79.2
Fasting blood glucose (mmol/L) - Mean (SE)	1321	4.9 (0.05)	4.6 (0.09)	4.6 - 5.1

* 95% confidence interval for the median

*SE* Standard error.

### IFG and diabetes prevalence and risk factors﻿

The prevalence of IFG and diabetes was 4.8% (95% CI: 3.6-6.3%) and 7.4% (95% CI: 5.5-10.0%), respectively. There were only slight differences in IFG prevalence across the assessed characteristics. The highest was between women and men, with women having nearly twofold higher prevalence than men [5.9% (95% CI: 4.3-8.0) vs 3.2% (95% CI: 2.0-4.9)]. The prevalence of diabetes differed across all characteristics assessed, although the CIs often overlapped. Overall, diabetes prevalence increases with age. The prevalence was almost twofold higher in the 45-64 age group (10.1, 95% CI: 6.8-14.8) than in the 25-44 age group (5.7, 95% CI: 4.1-8.1). Prevalence was higher in men (9.5, 95% CI: 5.6-15.5) than in women (6.0, 95% CI: 4.3-8.4) and in urban (10.2, 95% CI: 6.2-16.4) than in rural (6.1, 95% CI: 4.0-9.2) residents. There were also differences within subgroups; in the urban but not rural setting, there was a notable increase in prevalence with age, with a 10% difference between 25 and 44 and 45-64 groups [6,5% (95% CI: 3.9-10.7) vs 16% (95% CI: (9.1-26.7)] compared to only 2% in the rural setting [5.4% (95% CI: 3.3-8.8) vs 7.3% (95% CI: 4.3-12.1)]; likewise, females living in an urban setting aged 45-64 had a twofold higher prevalence compared to the 25-44 age group. Participants with formal education, primary level and above, had a higher prevalence than those with no education. Within the group with formal education, but not the group without formal education, differences were again noticeable between age groups; the older age group (45-65) had a twofold higher prevalence of diabetes than those in the 25-44 age group. The analysis also shows an increasing diabetes prevalence among men and women with overweight/obesity and central obesity (waist to height ratio) with age. Men and women with overweight/obesity aged 45-65 had a twofold higher prevalence of diabetes than those aged 25-44 [14.3% (95% CI: 9.5-20.8) vs 7.2% (95% CI: 3.9-12.9)], and participants with central obesity (WHtR ≥0.6) aged 45-65 had a sixfold higher prevalence of diabetes than those aged 25-44 [16.6% (95% CI: 9.6-27.1) vs 2.8%(95% CI: 0.8-8.9)]. (Table 2 and supplementary Tables S3, S4, and S5).

**Table 2:** Comparison of prevalence of diabetes in 2005 and 2015 by age group

	25 – 44			45 – 64			Total		
	2005	2015	Prevalence difference	2005	2015	Prevalence difference	2005	2015	Prevalence difference
	**% (95CI)**	**% (95CI)**	**% (95CI,** ***p*-value)**	**% (95CI)**	**% (95CI)**	**% (95CI,** ***p*-value)**	% (95CI)	% (95CI)	% (95CI, *p*-value)
All participants	2.3 (1.4-3.6)	5.7 (4.1-8.1)	3.5 (1.2-5.7, 0.002)	4.2 (2.7-6.4)	**10.1 (6.8-14.8)**	5.9 (1.6-10.2, 0.007)	2.9 (2.0-4.2)	**7.4 (5.5-10.0)**	**4.5 (2.1-7.0, < 0.001)**
Gender									
Female	1.6 (0.8-3.1)	**4.2 (2.6-6.8)**	2.6 (0.3-5.0, 0.025)	4.5 (2.4-8.6)	**9.8 (6.4-14.8)**	5.2 (0.2-10.3, 0.041)	2.4 (1.5-3.9)	6.0 (4.3-8.4)	3.6 (1.3-5.9, 0.002)
Male	3.3 (1.8-6.3)	8.6 (4.8-15.1)	5.3 (−0.1-10.6, 0.053)	3.8 (1.9-7.5)	10.5 (5.5-18.9)	6.7 (−0.2-13.6, 0.058)	3.5 (2.1-5.7)	**9.5 (5.6-15.5)**	**6.0 (0.9-11.0, 0.021)**
Place of residence									
Urban	4.1 (2.9-5.8)	**6.5 (3.9-10.7)**	2.4 (−1.2-5.9, 0.188)	7.4 (4.6-11.8)	**16.0 (9.1-26.7)**	8.6 (−0.7-17.9, 0.071)	5.2 (3.6-7.3)	**10.2 (6.2-16.4**)	**5.1 (−0.2-10.3, 0.058)**
Rural	1.3 (0.5-2.9)	5.4 (3.3-8.8)	4.1 (1.3-7.0, 0.004)	2.6 (1.3-5.2)	7.3 (4.3-12.1)	4.7 (0.5-8.8, 0.029)	1.7 (1.0-3.0)	6.1 (4.0-9.2)	4.4 (1.7-7.0, 0.001)
Education (years)									
None	0.8 (0.2-2.9)	5.2 (2.5-10.5)	4.3 (0.5-8.2, 0.026)	3.6 (1.5-8.3)	6.3 (3.4-11.5)	2.7 (−2.2-7.6, 0.279)	2.1 (1.1-4.0)	5.6 (3.4-9.2)	3.5 (0.4-6.6, 0.026)
1 - 5	1.8 (1.0-3.3)	**5.6 (3.3-9.3)**	3.8 (0.8-6.9, 0.014)	4.1 (2.1-8.0)	**12.6 (7.4-20.8)**	8.5 (1.4-15.5, 0.018)	2.5 (1.5-4.0)	8.4 (5.7-12.2)	**5.9 (2.5-9.3, 0.001)**
at least 6	5.3 (3.2-8.7)	6.5 (3.3-12.6)	1.3 (−3.9-6.4, 0.628)	6.8 (3.3-13.5)	**11.3 (6.0-20.2)**	4.5 (−3.8-12.8, 0.289)	5.5 (3.6-8.4)	7.8 (4.8-12.5)	2.3 (−2.1-6.7, 0.306)
Body mass index (kg/m2)									
< 25	1.3 (0.7-2.5)	5.2 (3.2-8.5)	3.9 (1.2-6.6, 0.004)	3.5 (2.0-6.1)	8.3 (4.5-14.8)	4.8 (−0.4-10.1, 0.072)	2.0 (1.3-3.1)	6.3 (4.0-9.9)	4.3 (1.4-7.3, 0.004)
≥ 25	7.7 (4.2-13.5)	7.2 (3.9-12.9)	−0.5 (−6.6-5.7, 0.880)	6.9 (3.8-12.2)	**14.3 (9.5-20.8)**	7.4 (0.6-14.2, 0.033)	7.4 (4.8-11.1)	10.6 (7.5-14.6)	3.2 (−1.4-7.8, 0.172)
Waist-to-height (WHtR) ratio									
< 0.5	1.5 (0.8-2.9)	4.7 (2.6-8.2)	3.2 (0.4-6.0, 0.026)	3.0 (1.4-6.1)	6.4 (3.5-11.3)	3.4 (−0.9-7.7, 0.117)	1.9 (1.2-3.1)	5.2 (3.2-8.6)	3.3 (0.6-6.1, 0.018)
≥ 0.5 - 0.6	4.5 (2.2-9.0)	8.8 (5.0-15.1)	4.4 (−1.4-10.1, 0.138)	5.6 (3.0-10.0)	**13.6 (7.5-23.5)**	8.0 (−0.4-16.5, 0.063)	4.9 (3.2-7.6)	11.0 (7.4-16.1)	**6.1 (1.3-10.9, 0.012)**
≥ 0.6	16.2 (6.9-33.8)	2.8 (0.8-8.9)	−13.5 (−26.8-0.1, 0.049)	15.4 (6.0-34.1)	**16.6 (9.6-27.1)**	1.2 (−14.8-17.1, 0.887)	15.8 (7.5-30.5)	10.6 (6.4-17.0)	−5.3 (−17.5-7.0, 0.400)

Using a log-binomial regression model, we included the overall data adjusted to gender, place of residence, age, education and BMI. In unadjusted analysis, men had a 67% (*p =* 0.057) higher diabetes prevalence than women, however, this effect loses statistical significance with adjusted estimates. Otherwise, findings show a statistically significant increase in diabetes prevalence with age, up to twice as high in the 55-64 year age group compared to the 25-34 age group (PR = 2.23, 95CI: 1.13-4.38) (Table 3).

**Table 3:** Regression model estimates for diabetes prevalence in STEPS 2015

Characteristic	Unadjusted^**a**^		Adjusted^**a,b**^	
	PR (95%CI)	***p***-value	PR (95%CI)	***p***-value
Gender				
Female	ref			
Male	1.67 (0.99 - 2.83)	0.057	1.57 (0.97 - 2.54)	0.071
Place of residence				
Rural	ref		ref	
Urban	1.49 (0.74 - 2.99)	0.264	1.50 (0.70 - 3.18)	0.297
Age (years)				
25 - 34	ref		ref	
35 - 44	1.30 (0.57 - 2.92)	0.535	1.15 (0.50 - 2.63)	0.736
45 - 54	1.41 (0.77 - 2.59)	0.267	1.28 (0.69 - 2.36)	0.431
55 - 64	2.40 (1.23 - 4.68)	0.012	2.23 (1.13 - 4.38)	0.023
Education (years)				
None	ref		ref	
1 - 5	1.17 (0.67 - 2.04)	0.584	1.14 (0.65 - 2.01)	0.651
at least 6	1.12 (0.53 - 2.34)	0.766	1.12 (0.53 - 2.33)	0.770
Body mass index (kg/m2)				
< 25	ref		ref	
≥ 25	1.43 (0.89 - 2.31)	0.146	1.29 (0.79 - 2.11)	0.305

^a^ Log-binomial model using survey design information with province level indicators

^b^ All covariates included

### Awareness and management of diabetes in rural and urban populations﻿

Only 10.1% (95% CI: 4.9 – 19.8) of study participants classified as diabetics were aware of their diagnosis and among these, less than half, 44% (95% CI: 12.1- 81.8) were taking glucose-lowering medicines. Additionally, approximately 80% (95% CI: 47.8 – 94.8) of participants also reported using non-pharmacological measures such as dietary changes (100%), exercise (48.2%), and weight loss (40%). Only a third of those who were aware of their diabetes status prior to the survey (33.3, 95% CI: 5.7 – 80.6) were controlled (had measurements below the WHO definition of diabetes used in this study).

### Comparison of diabetes prevalence and awareness between 2005 and 2015

#### Study samples compared characteristics

The study samples of 2005 and 2015 were comparable in terms of sex, area of residence distribution, and level of education, however there were some differences in the age group distribution. For example, there was a 6% decrease in the 25-34 age group and a 4% increase in the 55-64 age group, which resulted in a two-year increase in the mean age of the 2015 survey participants, from 39.4 (2005) to 41.4 years (2015). Although mean BMI [9] did not differ in study samples, abdominal circumference increased by 3.6 cm for women and 2.2 cm for men, and mean FBG increased from 3.8 mmol/L in 2005 to 4.6 mmol/L in 2015 [9]. It was conducted age-sex standardized diabetes and IFG prevalence for the two survey populations (2005 and 2015) using the 2017 census population to allow comparability. Apart from small numeric changes, the results did not change substantially. (Table 4 and Supplementary Table S6).

**Table 4:** Mozambique age -sex standardized prevalence of Diabetes among people between the ages 25 and 64 years, in 2005 and 2015

	2005^**a**^	2015^**a**^	Prevalence difference	***p***-value
Characteristic	% (95CI)	% (95CI)	% (95CI)	
Overall	3.0 (2.1; 4.2)	7.3 (5.3; 9.9)	4.3 (1.8; 6.8)	< 0.001
Gender				
Female	2.5 (1.6; 3.8)	5.8 (4.2; 8.2)	3.4 (1.1; 5.6)	0.003
Male	3.5 (2.1; 5.7)	8.9 (5.3; 14.7)	5.4 (0.5; 10.3)	0.031
Place of residence				
Urban	5.1 (3.5; 7.3)	9.3 (5.8; 14.5)	4.2 (−0.4; 8.9)	0.077
Rural	1.8 (1.1; 3.1)	6.3 (4.0; 9.9)	4.5 (1.4; 7.6)	0.004
Education (years)				
None	2.1 (1.1; 4.0)	5.8 (3.5; 9.5)	3.7 (0.5; 6.9)	0.024
1 - 5	2.4 (1.5; 3.8)	7.9 (5.3; 11.5)	5.4 (2.2; 8.6)	0.001
at least 6	5.6 (3.5; 8.9)	7.8 (4.2; 14.2)	2.2 (−3.3; 7.7)	0.433
Body mass index (kg/m2)				
< 25	2.1 (1.4; 3.0)	6.2 (3.8; 9.9)	4.1 (1.1; 7.2)	0.008
≥ 25	7.8 (5.1; 11.7)	10.5 (7.2; 15.0)	2.7 (−2.3; 7.7)	0.292
Waist-to-height (WHtR) ratio				
< 0.5	2.0 (1.2; 3.1)	5.4 (3.1; 9.4)	3.5 (0.3; 6.6)	0.031
≥ 0.5 - 0.6	5.6 (3.5; 8.9)	10.5 (7.1; 15.3)	4.9 (0.1; 9.7)	0.048
≥ 0.6	15.1 (7.8; 27.4)	11.1 (6.7; 17.9)	−4.0 (−15.1; 7.1)	0.476

^**a**^ The prevalence is age and sex standardized to 2017 Mozambique’s Population Census

### Differences in the prevalence of IFG and diabetes between 2005 and 2015

The IFG prevalence more than doubled between 2005 and 2015 (2.8% 95CI: 1.1 – 4.6, *p =* 0.001) from 2 to 4.8%. This increase in the prevalence resulted from the combination of a 4% (*p =* 0.000) increase in women, a 4.9% (*p =* 0.001) increase in the urban residents, and a 5.1% (*p =* 0.003) and 5.8% (*p =* 0.123) increase in the population with overweight/obesity and central obesity, respectively.

As seen in Table 2, overall, between 2005 and 2015, the prevalence of diabetes doubled (*p =* 0.001), with a threefold increase in men (*p =* 0.021), people living in rural areas (*p =* 0.001) and with primary level education (*p =* 0.001) and a twofold increase in women (*p =* 0.002) and people living in urban areas (*p =* 0.058). No difference was observed concerning obesity levels. In addition, diabetes prevalence in men increased approximately twofold compared to women and with the number of years of education. For all sub-groups of analysis, the prevalence of diabetes has increased with age, Supplementary Fig. 2, and despite the similar trend in both years, the 2015 curve is steeper. This trend was not affected by the state of obesity or not. Refer to Table 3 for more details. Similarly, participants with central obesity (WHtR) had a higher prevalence of diabetes than those with WHtR < 0.5.

The proportion of people aware of their diabetes status has decreased by 3%, and about 50% of those who were aware were taking glucose-lowering drugs in 2015 compared to 2005 [9]. However, care-seeking with traditional healers remained unchanged compared to 2005 [9].

## Discussion

Roughly 7% of adults aged 25 to 64 years had type 2 diabetes in Mozambique in 2015. This is more than twofold higher than the reported prevalence in 2005. Despite women having more risk factors, men were more affected by type 2 diabetes [24]. Likewise, older age groups and residents in urban areas were more affected than their younger and rural counterparts, although a significant increase was also observed in rural young adults from 2005 to 2015. The diabetes care cascade has worsened since 2005, with 3% fewer diabetic patients aware of their status and 50% fewer on treatment among those aware [9].

The rise in prevalence found in this study is not limited to Mozambique in the SSA region. Other studies measured diabetes prevalence over time and reported rising results, albeit in smaller proportions. For example, a STEPS survey conducted in Zambia in 2008 and 2017 reported a rise from 4.0% [12] to 6.2% [13]. Another study measured diabetes prevalence in a black urban population in South Africa, and reported an increase from 8% in 1990 to 12.2% in 2008/09 [25]. A faster pace of urbanization in Mozambique, over the period of study, may explain the faster increase in diabetes prevalence seen in Zambia and South Africa, both of which had attained greater urbanization by the first survey (2005) in Mozambique.

Participants aged 45-64 years showed a statistically significant higher type 2 diabetes prevalence than that found for the 25-44 years age group. This finding is in keeping with the growing trend in type 2 diabetes observed in other settings [26, 27].

The rising diabetes prevalence found in this study may be attributed to some drivers. One of the most remarkable was overweight and obesity. Total obesity measured by the BMI ≥ 25 was present in 30% of the study sample and 48% of participants with diabetes (data not shown). However, a steep increase in diabetes prevalence was observed in the subgroup of people with BMI < 25. A similar finding was reported in Malawi by Price and colleagues [28] and contrasted what is reported in HIC, where only about a fifth of patients with diabetes have a normal BMI [29]. This finding suggests that other important factors, which are not captured by this survey, may contribute to the occurrence of diabetes besides obesity it this setting. A study done by Fontes and colleagues [24] in Mozambique reported a higher prevalence of obesity in young adults. However, in contrast, we found that obesity was twofold higher in older age groups (45-64 years).

Participants living in urban areas were 50% more likely to have diabetes than those in rural areas. In another study using the same data [24], this was attributed to a decline in physical activity, greater availability and consumption of high-calorie foods, and declined consumption of fruits and vegetables. The effect of urbanization as well as changes in diet profile and physical inactivity, tend to be more pronounced in men in settings like Mozambique. The literature suggests that men are more prone to work outside the home, to have most of their meals there, and own more motor vehicles [24, 28]. Similar findings on urban-rural differences were reported by studies done elsewhere (Ethiopia (4.6% vs 2.0%) [30], Kenya (4-11% vs 3%) [31]). Although urban-rural differences were observed in similar proportions between 2005 and 2015, we observed an abrupt and significant increase in the prevalence of diabetes in younger (25-44 years) rural dwellers. The reason for this increase is not clear.

While not statistically significant, diabetes prevalence was almost 60% higher in men than in women, which is consistent with the global figures for diabetes sex-distribution [2].

Review of the diabetes care cascade in our sample has revealed that 90% of participants were unaware of their diabetes status, with less than half of those who were aware taking glucose-lowering drugs. Among those taking medication, only one third reported having their diabetes controlled. Mozambique’s is not unique in this sense. These figures are in keeping with an analysis of 12 SSA countries conducted by Atun and colleagues [5]. They found a total of 89% of drop-offs in care, including 50% loss to diagnose, 13% not aware of their diabetes status, 20% not receiving any advice on lifestyle modification, and 6% not starting medication [5]. In fact, the actual burden of diabetes, and other relevant information for appropriate management of NCDs in sub-Saharan Africa, including in Mozambique, are still inadequately known [5–7].

This study had a number of limitations. Only 60% of the total population sampled complied with the 12-hour fasting requirement. Our sample population was older, had a higher proportion reporting being from rural areas, and had overall larger waist circumferences. This may have biased our findings, potentially resulting in an overestimation of the burden of risk factors and disease associated with increasing age and central obesity on the one hand and an underestimation of the disease prevalence associated with an urban residence. These aspects can lead to an underestimation of the actual disease burden in our total sample. A further limitation was our use of capillary blood samples and POC glucose meters rather than laboratory blood glucose estimations to determine the level of blood glucose [32]. Studies suggest that using this type of sample is associated with an underestimation of the actual blood glucose concentration [33]. Nevertheless, this remains the most common way that glycaemia is evaluated in this type of population-based study. Finally, the estimates used for comparison between 2005 and 2015 did not consider the changes in the overall population structure, which could lead to overestimating the level of increased disease burden found between the two periods. In addition, the precision of estimates, such as those in the diabetes cascade, was affected by the sample size.

The increase in prevalence of type 2 diabetes as reported here is concerning. Despite, Mozambique’s prioritization of NCDs in several national policy documents [34], program implementation of such policies has not achieved their desired results. For example, in 2021, Cárdenas and colleagues found that the primary health care (PHC) was inadequately prepared to offer NCD related services in three countries, including Mozambique [35]. As the healthcare services stand now [5], they are far from meeting the actual needs of diabetes related services and miss many opportunities for preventing and controlling them. If appropriate measures are not duly taken diabetes management costs to the health system will continue to pile up and further aggravate the already challenged health systems [5, 36].

## Conclusion

The rising prevalence of diabetes and the population’s low rates of awareness about their disease and its subsequent management are concerning. This calls for urgent adaptation of health service delivery, mainly at the primary health care level, to improve diabetes detection and management and to promote community-based initiatives to improve diabetes related health education and prevention.

## Supplementary Information

**Additional file 1.**

**Additional file 2.**

## Data Availability

The datasets used during the current study are available from the corresponding author on reasonable request.

## Abbreviations

BMI: Body mass index
CI: Confidence interval
CSPRO: Census and Survey Processing System
FBG: Fasting blood glucose
HIC: High-income countries
IFG: Impaired fasting glucose
INE: National Statistics Institute
LMIC: Low and middle-income countries
MOH: Ministry of Health
NCDs: Noncommunicable diseases
OAD: Oral antidiabetic drug
PD: Prevalence difference
PHC: Primary health care
SSA: Sub-Saharan Africa
STEPS: Stepwise approach to NCD risk factor surveillance
WHO: World Health Organization
WHtH: Waist-to-hip ratio
WHtR: Waist-to-height ratio
