# Supplementary material for: Changes in prevalence and the cascade of care for type 2 diabetes over ten years (2005-2015): results of two nationally representative surveys in Mozambique
Source: BMC Public Health. 2022 Nov 25;22:2174. doi: 10.1186/s12889-022-14595-7 (PMC9701039; doi:10.1186/s12889-022-14595-7)
Supplement: Supplementary file 1 — Additional file 1. [file 12889_2022_14595_MOESM1_ESM.docx]

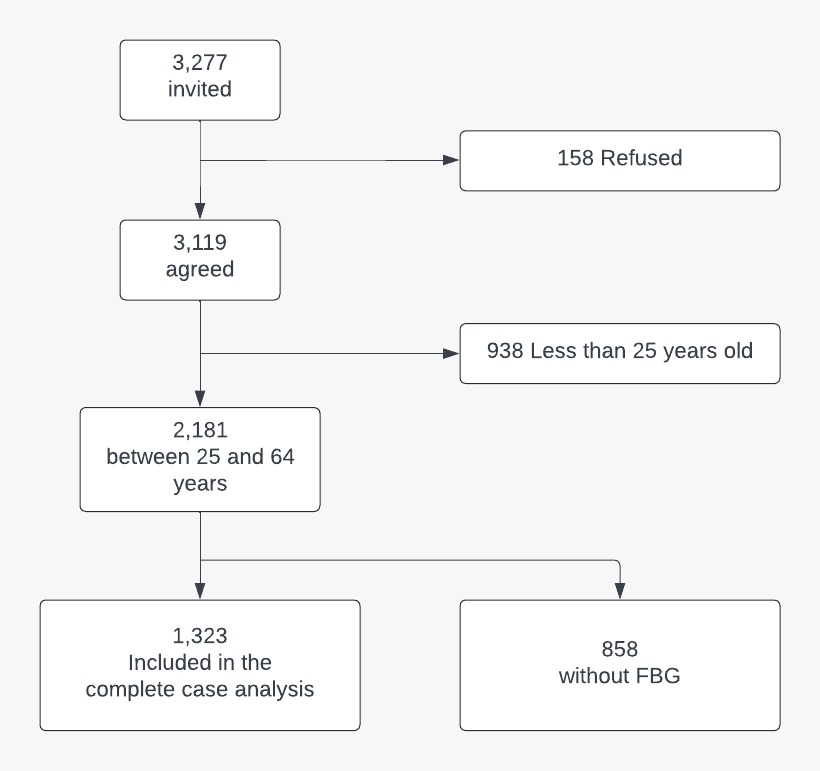


**Figure 1 - Flowchart of the participants included and excluded in the study sample**


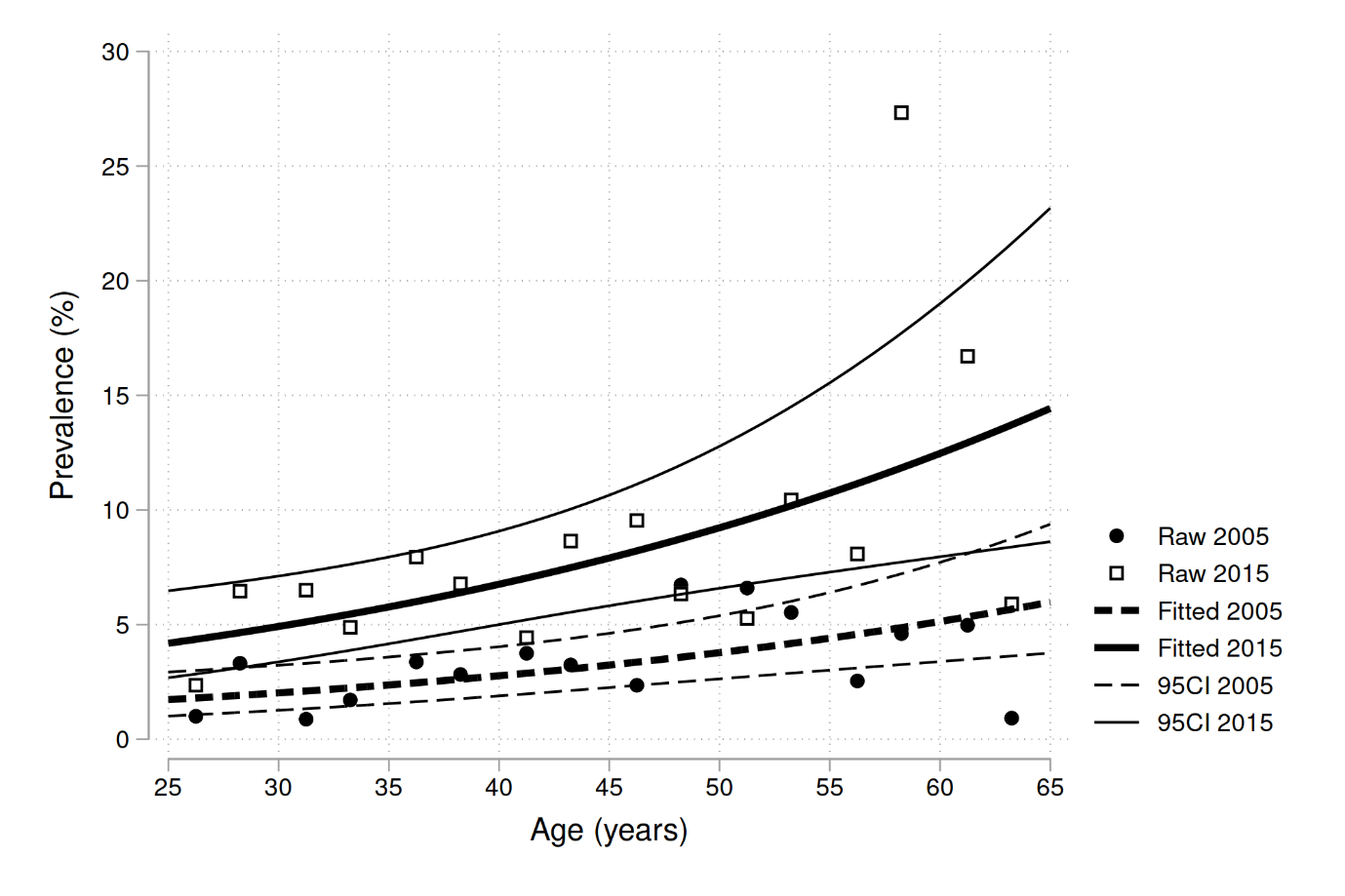


**Figure 2 - 2005 and 2015 comparison of type 2 diabetes prevalence trend in adult Mozambican population from 25 to 65 years.**

The points are raw prevalence for each 2.5 years age group. The lines are fitted from a logistic regression on the aggregated dataset.
