# Supplementary material for: Changes in prevalence and the cascade of care for type 2 diabetes over ten years (2005-2015): results of two nationally representative surveys in Mozambique
Source: BMC Public Health. 2022 Nov 25;22:2174. doi: 10.1186/s12889-022-14595-7 (PMC9701039; doi:10.1186/s12889-022-14595-7)
Supplement: Supplementary file 2 — Additional file 2. [file 12889_2022_14595_MOESM2_ESM.docx]

Table S1 – Comparison of diabetes prevalence estimates between the complete case analysis and multiple imputations based estimates

|  | **2015 Complete Case Analysis** | **2015 MICE Analysis** |
| --- | --- | --- |
|  | **% (95CI)** | **% (95CI)** |
| All participants | 7.4 (5.5-10.0) | 8.3 (5.7-10.9) |
| Gender |  |  |
| Female | 6.0 (4.3-8.4) | 7.2 (4.8-9.6) |
| Male | 9.5 (5.6-15.5) | 10.0 (5.0-15.0) |
| Place of residence |  |  |
| Urban | 10.2 (6.2-16.4) | 11.1 (6.3-15.9) |
| Rural | 6.1 (4.0-9.2) | 7.0 (4.0-10.1) |
| Education (years) |  |  |
| None | 5.6 (3.4-9.2) | 5.9 (3.1-8.7) |
| 1 - 5 | 8.4 (5.7-12.2) | 10.0 (5.9-14.0) |
| at least 6 | 7.8 (4.8-12.5) | 8.1 (4.4-11.9) |
| Body mass index (kg/m2) |  |  |
| < 25 | 6.3 (4.0-9.9) | 7.3 (3.9-10.6) |
| ≥ 25 | 10.6 (7.5-14.6) | 11.3 (7.5-15.0) |
| Waist-to-height (WHtR) ratio |  |  |
| < 0.5 | 5.2 (3.2-8.6) | 6.2 (2.9-9.5) |
| ≥ 0.5 - 0.6 | 11.0 (7.4-16.1) | 11.7 (7.3-16.1) |
| ≥ 0.6 | 10.6 (6.4-17.0) | 11.1 (5.6-16.5) |
| Waist-to-hip (WHtH) ratio* |  |  |
| < 0.9 | 6.9 (4.6-10.2) | 7.6 (4.7-10.5) |
| ≥ 0.9 | 8.2 (5.8-11.4) | 9.2 (6.0-12.4) |

* Among males cut-off Is 0.90 and females is 0.85

MICE - multiple imputation by chained equations

Table S2 – Comparison of IFG prevalence estimates between the complete case analysis and multiple imputations based estimates

|  | **2015 Complete Case Analysis** | **2015 MICE Analysis** |
| --- | --- | --- |
|  | **% (95CI)** | **% (95CI)** |
| All participants | 4.8 (3.6-6.3) | 4.8 (3.4-6.2) |
| Gender |  |  |
| Female | 5.9 (4.3-8.0) | 5.5 (3.8-7.2) |
| Male | 3.2 (2.0-4.9) | 3.8 (2.1-5.5) |
| Place of residence |  |  |
| Urban | 6.7 (4.5-9.9) | 7.5 (4.5-10.5) |
| Rural | 3.9 (2.7-5.7) | 3.6 (2.2-4.9) |
| Education (years) |  |  |
| None | 4.8 (3.0-7.7) | 4.7 (2.4-7.0) |
| 1 - 5 | 4.6 (3.2-6.6) | 4.6 (3.0-6.2) |
| at least 6 | 5.5 (3.3-8.9) | 5.8 (2.8-8.7) |
| Body mass index (kg/m2) |  |  |
| < 25 | 4.3 (3.0-5.9) | 3.9 (2.4-5.3) |
| ≥ 25 | 7.1 (4.5-11.0) | 7.5 (4.5-10.5) |
| Waist-to-height (WHtR) ratio |  |  |
| < 0.5 | 4.4 (3.1-6.4) | 4.0 (2.4-5.6) |
| ≥ 0.5 - 0.6 | 5.2 (3.4-7.8) | 5.5 (3.5-7.5) |
| ≥ 0.6 | 8.5 (3.6-18.9) | 8.1 (1.3-14.9) |
| Waist-to-hip (WHtH) ratio* |  |  |
| < 0.9 | 4.3 (2.9-6.3) | 4.5 (2.8-6.1) |
| ≥ 0.9 | 5.9 (4.0-8.6) | 5.2 (3.3-7.2) |

* Among males cut-off Is 0.90 and females is 0.85

MICE - multiple imputation by chained equations

Table S3 – Overall IFG prevalence in percentages in years 2005 and 2015, per age categories

|  | **25-44** | | | **45-64** | | | **Total** | | |
| --- | --- | --- | --- | --- | --- | --- | --- | --- | --- |
|  | **2005** | **2015** | **Prevalence difference** | **2005** | **2015** | **Prevalence difference** | **2005** | **2015** | **Prevalence difference** |
|  | **% (95CI)** | **% (95CI)** | **% (95CI, p-value)** | **% (95CI)** | **% (95CI)** | **% (95CI, p-value)** | **% (95CI)** | **% (95CI)** | **% (95CI, p-value)** |
| All participants | 1.2 (0.7-2.2) | 4.2 (2.9-6.0) | 3.0 (1.3-4.6, 0.000) | 3.5 (1.1-10.7) | 5.8 (3.9-8.6) | 2.3 (-2.2-6.9, 0.314) | 2.0 (1.1-3.5) | 4.8 (3.6-6.3) | 2.8 (1.1-4.6, 0.001) |
| Gender |  |  |  |  |  |  |  |  |  |
| Female | 1.3 (0.7-2.3) | 5.1 (3.4-7.6) | 3.9 (1.7-6.1, 0.001) | 3.3 (0.9-10.9) | 7.5 (4.7-11.8) | 4.2 (-1.1-9.5, 0.121) | 1.9 (1.1-3.2) | 5.9 (4.3-8.0) | 4.0 (2.0-6.1, 0.000) |
| Male | 1.2 (0.5-2.7) | 2.5 (1.3-4.7) | 1.3 (-0.6-3.1, 0.192) | 3.6 (1.2-10.7) | 4.0 (2.1-7.4) | 0.4 (-4.3-5.0, 0.881) | 2.1 (1.0-4.4) | 3.2 (2.0-4.9) | 1.1 (-1.0-3.1, 0.320) |
| Place of residence |  |  |  |  |  |  |  |  |  |
| Urban | 1.7 (0.8-3.5) | 6.5 (4.2-9.9) | 4.8 (1.8-7.8, 0.002) | 2.2 (1.1-4.4) | 7.1 (3.7-13.3) | 4.9 (0.1-9.7, 0.045) | 1.8 (1.0-3.3) | 6.7 (4.5-9.9) | 4.9 (2.0-7.7, 0.001) |
| Rural | 1.0 (0.5-2.1) | 3.2 (1.8-5.6) | 2.2 (0.3-4.1, 0.027) | 4.1 (0.9-16.1) | 5.2 (3.1-8.6) | 1.1 (-5.3-7.5, 0.737) | 2.0 (0.9-4.7) | 3.9 (2.7-5.7) | 1.9 (-0.3-4.2, 0.095) |
| Education (years) |  |  |  |  |  |  |  |  |  |
| None | 0.7 (0.2-1.9) | 4.1 (1.9-8.4) | 3.4 (0.3-6.5, 0.030) | 1.1 (0.4-2.7) | 6.0 (3.5-10.1) | 4.9 (1.6-8.2, 0.004) | 0.9 (0.4-1.9) | 4.8 (3.0-7.7) | 4.0 (1.7-6.3, 0.001) |
| 1 - 5 | 1.6 (0.9-2.8) | 4.1 (2.3-7.2) | 2.5 (0.0-5.0, 0.046) | 6.2 (1.5-22.0) | 5.3 (3.0-9.2) | -0.8 (-9.6-8.0, 0.854) | 2.9 (1.4-6.0) | 4.6 (3.2-6.6) | 1.7 (-1.0-4.4, 0.226) |
| at least 6 | 1.0 (0.4-2.5) | 4.7 (2.7-8.1) | 3.7 (1.0-6.4, 0.008) | 2.3 (0.7-7.3) | 7.5 (2.8-18.6) | 5.3 (-2.3-12.9, 0.173) | 1.2 (0.6-2.5) | 5.5 (3.3-8.9) | 4.2 (1.4-7.1, 0.004) |
| Body mass index (kg/m2) |  |  |  |  |  |  |  |  |  |
| < 25 | 1.1 (0.6-1.9) | 3.6 (2.2-5.8) | 2.5 (0.7-4.4, 0.007) | 3.9 (1.0-13.6) | 5.5 (3.3-8.8) | 1.6 (-4.1-7.3, 0.580) | 1.9 (0.9-4.1) | 4.3 (3.0-5.9) | 2.3 (0.3-4.4, 0.023) |
| ≥ 25 | 2.0 (0.9-4.6) | 7.3 (4.1-12.7) | 5.3 (0.9-9.7, 0.018) | 2.1 (1.0-4.3) | 6.9 (3.6-12.7) | 4.8 (0.2-9.3, 0.039) | 2.1 (1.2-3.6) | 7.1 (4.5-11.0) | 5.1 (1.7-8.4, 0.003) |
| Waist-to-height ratio |  |  |  |  |  |  |  |  |  |
| < 0.5 | 1.3 (0.7-2.4) | 3.7 (2.2-6.2) | 2.4 (0.3-4.5, 0.024) | 4.4 (1.1-15.4) | 6.0 (3.5-10.1) | 1.6 (-4.9-8.1, 0.628) | 2.2 (1.1-4.6) | 4.4 (3.1-6.4) | 2.2 (-0.1-4.5, 0.056) |
| ≥ 0.5 - 0.6 | 1.1 (0.4-3.5) | 4.6 (2.7-7.8) | 3.5 (0.8-6.2, 0.011) | 1.1 (0.4-2.7) | 5.9 (3.2-10.4) | 4.8 (1.2-8.3, 0.008) | 1.1 (0.5-2.4) | 5.2 (3.4-7.8) | 4.1 (1.8-6.4, 0.000) |
| ≥ 0.6 | 0.7 (0.1-5.1) | 12.2 (4.3-30.1) | 11.6 (-0.5-23.6, 0.060) | 4.6 (1.5-13.1) | 5.6 (1.9-15.4) | 1.0 (-6.7-8.7, 0.796) | 2.7 (1.0-6.8) | 8.5 (3.6-18.9) | 5.8 (-1.7-13.3, 0.129) |
| Waist-to-hip ratio* |  |  |  |  |  |  |  |  |  |
| < 0.9 | - | 3.9 (2.3-6.5) | - | - | 5.2 (2.8-9.5) | - | - | 4.3 (2.9-6.3) | - |
| ≥ 0.9 | - | 5.4 (3.2-8.9) | - | - | 6.5 (3.7-11.0) | - | - | 5.9 (4.0-8.6) | - |
| * Among males cut-off Is 0.90 and females is 0.85 | | |  |  |  |  |  |  |  |

Table S4 – Urban diabetes prevalence in percentages in years 2005 and 2015, per age categories

|  | **25-44** | | | **45-64** | | | **Total** | | |
| --- | --- | --- | --- | --- | --- | --- | --- | --- | --- |
|  | **2005** | **2015** | **Prevalence difference** | **2005** | **2015** | **Prevalence difference** | **2005** | **2015** | **Prevalence difference** |
|  | **% (95CI)** | **% (95CI)** | **% (95CI, p-value)** | **% (95CI)** | **% (95CI)** | **% (95CI, p-value)** | **% (95CI)** | **% (95CI)** | **% (95CI, p-value)** |
| All participants | 4.1 (2.9-5.8) | 6.5 (3.9-10.7) | 2.4 (-1.2-5.9, 0.188) | 7.4 (4.6-11.8) | 16.0 (9.1-26.7) | 8.6 (-0.7-17.9, 0.071) | 5.2 (3.6-7.3) | 10.2 (6.2-16.4) | 5.1 (-0.2-10.3, 0.058) |
| Gender |  |  |  |  |  |  |  |  |  |
| Female | 4.0 (2.2-7.2) | 5.3 (2.9-9.7) | 1.3 (-2.7-5.3, 0.513) | 6.9 (3.1-14.7) | 13.7 (7.7-23.2) | 6.7 (-2.5-15.9, 0.152) | 4.9 (3.1-7.5) | 8.2 (5.1-13.0) | 3.3 (-1.0-7.7, 0.133) |
| Male | 4.3 (1.7-10.4) | 9.3 (3.8-20.9) | 5.0 (-3.8-13.8, 0.266) | 8.0 (3.6-17.1) | 19.2 (7.4-41.4) | 11.2 (-6.6-29.0, 0.218) | 5.5 (3.3-9.3) | 14.0 (5.7-30.3) | 8.5 (-3.6-20.5, 0.169) |
| Education (years) |  |  |  |  |  |  |  |  |  |
| None | 2.3 (0.6-8.4) | 9.9 (2.8-30.0) | 7.7 (-4.7-20.0, 0.224) | 6.5 (1.5-23.4) | 9.5 (3.5-23.5) | 3.0 (-9.7-15.8, 0.639) | 4.6 (1.7-12.0) | 9.7 (4.2-21.0) | 5.2 (-3.9-14.2, 0.261) |
| 1 - 5 | 2.3 (1.2-4.4) | 7.3 (3.4-15.1) | 5.0 (-0.7-10.7, 0.084) | 7.1 (3.4-14.4) | 19.8 (9.1-38.0) | 12.7 (-2.5-27.8, 0.101) | 4.0 (2.4-6.6) | 12.9 (7.3-21.8) | 8.9 (1.6-16.1, 0.017) |
| at least 6 | 6.3 (3.7-10.4) | 4.2 (2.0-8.5) | -2.1 (-6.4-2.3, 0.355) | 8.3 (4.0-16.4) | 14.6 (7.8-25.9) | 6.3 (-4.2-16.9, 0.240) | 6.6 (4.3-10.1) | 7.1 (4.3-11.5) | 0.5 (-3.9-4.9, 0.822) |
| Body mass index (kg/m2) |  |  |  |  |  |  |  |  |  |
| < 25 | 1.8 (0.9-3.6) | 6.2 (2.8-13.0) | 4.4 (-0.5-9.3, 0.077) | 6.4 (3.0-13.1) | 14.2 (5.0-34.3) | 7.9 (-6.8-22.5, 0.291) | 3.0 (1.8-5.1) | 9.2 (3.8-20.5) | 6.2 (-1.7-14.0, 0.123) |
| ≥ 25 | 10.3 (5.9-17.4) | 7.4 (3.7-14.3) | -2.9 (-10.3-4.5, 0.440) | 9.2 (5.1-15.9) | 17.9 (11.2-27.4) | 8.7 (-0.8-18.2, 0.072) | 9.9 (6.7-14.3) | 12.0 (8.1-17.5) | 2.1 (-3.8-8.0, 0.483) |
| Waist-to-height ratio |  |  |  |  |  |  |  |  |  |
| < 0.5 | 2.3 (1.3-4.1) | 8.8 (4.7-15.7) | 6.5 (1.0-11.9, 0.019) | 5.4 (1.9-14.4) | 6.8 (2.9-15.4) | 1.5 (-6.4-9.4, 0.717) | 3.1 (1.8-5.2) | 8.2 (4.7-13.9) | 5.1 (0.5-9.8, 0.032) |
| ≥ 0.5 - 0.6 | 6.8 (3.0-14.6) | 4.3 (1.8-9.7) | -2.5 (-8.9-3.9, 0.443) | 6.6 (3.1-13.5) | 22.5 (10.5-41.7) | 15.9 (-0.4-32.2, 0.055) | 6.7 (4.2-10.5) | 13.3 (6.7-24.6) | 6.6 (-2.6-15.7, 0.158) |
| ≥ 0.6 | 22.0 (10.3-41.0) | 4.3 (1.2-13.6) | -17.7 (-33.8--1.7, 0.030) | 19.8 (8.4-39.9) | 16.4 (8.5-29.4) | -3.4 (-21.9-15.2, 0.722) | 20.8 (10.3-37.3) | 10.7 (5.7-19.1) | -10.1 (-24.8-4.7, 0.182) |
| Waist-to-hip ratio* |  |  |  |  |  |  |  |  |  |
| < 0.9 | - | 8.2 (4.2-15.6) | - | - | 17.1 (8.5-31.3) | - | - | 10.9 (5.8-19.5) | - |
| ≥ 0.9 | - | 4.3 (2.0-9.2) | - | - | 15.4 (8.5-26.4) | - | - | 10.1 (6.2-16.2) | - |
| * Among males cut-off Is 0.90 and females is 0.85 | | |  |  |  |  |  |  |  |

Table S5 – Rural diabetes prevalence in percentages in years 2005 and 2015, per age categories

|  | **25-44** | | | **45-64** | | | **Total** | | |
| --- | --- | --- | --- | --- | --- | --- | --- | --- | --- |
|  | **2005** | **2015** | **Prevalence difference** | **2005** | **2015** | **Prevalence difference** | **2005** | **2015** | **Prevalence difference** |
|  | **% (95CI)** | **% (95CI)** | **% (95CI, p-value)** | **% (95CI)** | **% (95CI)** | **% (95CI, p-value)** | **% (95CI)** | **% (95CI)** | **% (95CI, p-value)** |
| All participants | 1.3 (0.5-2.9) | 5.4 (3.3-8.8) | 4.1 (1.3-7.0, 0.004) | 2.6 (1.3-5.2) | 7.3 (4.3-12.1) | 4.7 (0.5-8.8, 0.029) | 1.7 (1.0-3.0) | 6.1 (4.0-9.2) | 4.4 (1.7-7.0, 0.001) |
| Gender |  |  |  |  |  |  |  |  |  |
| Female | 0.4 (0.1-1.9) | 3.7 (1.8-7.6) | 3.3 (0.5-6.0, 0.020) | 3.3 (1.1-9.2) | 7.5 (3.8-14.4) | 4.2 (-1.8-10.3, 0.172) | 1.2 (0.5-3.1) | 4.9 (2.9-8.1) | 3.6 (0.9-6.4, 0.009) |
| Male | 2.8 (1.2-6.3) | 8.4 (3.9-17.3) | 5.6 (-1.0-12.3, 0.098) | 1.8 (0.6-5.1) | 7.0 (3.2-14.4) | 5.2 (-0.3-10.7, 0.066) | 2.4 (1.1-4.9) | 7.8 (4.0-14.5) | 5.4 (0.1-10.6, 0.045) |
| Education (years) |  |  |  |  |  |  |  |  |  |
| None | 0.6 (0.1-3.3) | 4.2 (1.7-9.8) | 3.5 (-0.2-7.3, 0.065) | 2.9 (1.0-8.3) | 5.4 (2.4-11.6) | 2.5 (-2.8-7.7, 0.356) | 1.6 (0.8-3.5) | 4.6 (2.5-8.5) | 3.0 (-0.1-6.1, 0.060) |
| 1 - 5 | 1.6 (0.6-3.8) | 5.0 (2.5-9.9) | 3.4 (-0.3-7.2, 0.068) | 2.3 (0.7-7.1) | 9.2 (4.7-17.4) | 7.0 (0.4-13.6, 0.038) | 1.8 (0.8-3.6) | 6.6 (3.8-11.3) | 4.9 (1.1-8.6, 0.012) |
| at least 6 | 2.1 (0.5-7.6) | 10.9 (3.8-27.6) | 8.8 (-2.4-20.0, 0.124) | - | 3.9 (0.5-23.5) | - | 1.8 (0.5-6.6) | 9.2 (3.3-22.7) | 7.4 (-1.8-16.5, 0.113) |
| Body mass index (kg/m2) |  |  |  |  |  |  |  |  |  |
| < 25 | 1.2 (0.5-2.9) | 4.9 (2.6-9.2) | 3.8 (0.5-7.1, 0.023) | 2.6 (1.2-5.6) | 6.2 (3.2-11.8) | 3.6 (-0.9-8.1, 0.118) | 1.6 (0.9-2.9) | 5.4 (3.1-9.2) | 3.8 (0.7-6.8, 0.016) |
| ≥ 25 | 2.6 (0.7-9.9) | 7.0 (2.1-20.3) | 4.3 (-4.3-12.9, 0.327) | 2.5 (0.4-15.5) | 11.0 (5.4-21.2) | 8.5 (-0.4-17.4, 0.061) | 2.6 (0.9-7.0) | 9.0 (4.7-16.5) | 6.5 (0.3-12.6, 0.041) |
| Waist-to-height ratio |  |  |  |  |  |  |  |  |  |
| < 0.5 | 1.1 (0.4-3.2) | 3.3 (1.2-8.5) | 2.2 (-1.2-5.5, 0.212) | 2.2 (0.8-5.9) | 6.2 (3.0-12.7) | 4.0 (-1.0-9.0, 0.117) | 1.5 (0.7-3.0) | 4.3 (2.0-9.1) | 2.8 (-0.6-6.2, 0.105) |
| ≥ 0.5 - 0.6 | 2.2 (0.8-6.3) | 11.4 (5.9-20.9) | 9.2 (1.6-16.8, 0.017) | 4.6 (1.6-12.2) | 7.3 (3.5-14.4) | 2.7 (-4.1-9.5, 0.440) | 3.2 (1.4-7.4) | 9.6 (5.8-15.6) | 6.4 (1.0-11.8, 0.021) |
| ≥ 0.6 | 5.4 (0.4-41.6) | - | - | - | 16.8 (6.5-36.9) | - | 3.2 (0.4-23.1) | 10.3 (4.2-23.2) | 7.1 (-4.0-18.2, 0.211) |
| Waist-to-hip ratio* |  |  |  |  |  |  |  |  |  |
| < 0.9 | - | 4.6 (2.5-8.4) | - | - | 6.0 (2.8-12.6) | - | - | 5.1 (2.8-9.0) | - |
| ≥ 0.9 | - | 6.2 (2.9-12.7) | - | - | 8.7 (4.7-15.4) | - | - | 7.3 (4.4-11.7) | - |
| * Among males cut-off Is 0.90 and females is 0.85 | | |  |  |  |  |  |  |  |

Table S6 – Mozambique age -sex standardized prevalence of IFG among people between the ages 25 and 64 years, in 2005 and 2015

|  | **2005** | **2015** | **Prevalence difference** | **p-value** |
| --- | --- | --- | --- | --- |
| **Characteristic** | **% (95CI)** | **% (95CI)** | **% (95CI)** |  |
| Overall | 1.9 (1.1 ; 3.4) | 4.5 (3.4 ; 6.1) | 2.6 (0.9 ; 4.4) | 0.003 |
| Gender |  |  |  |  |
| Female | 1.9 (1.1 ; 3.1) | 5.9 (4.2 ; 8.1) | 4.0 (1.9 ; 6.1) | < 0.001 |
| Male | 2.0 (0.9 ; 4.1) | 3.0 (1.9 ; 4.7) | 1.0 (-1.0 ; 3.0) | 0.319 |
| Place of residence |  |  |  |  |
| Urban | 1.9 (1.0 ; 3.4) | 6.9 (4.6 ; 10.4) | 5.1 (2.0 ; 8.1) | 0.001 |
| Rural | 2.0 (0.8 ; 4.5) | 3.5 (2.3 ; 5.2) | 1.5 (-0.7 ; 3.7) | 0.176 |
| Education (years) |  |  |  |  |
| None | 0.9 (0.4 ; 2.0) | 4.6 (2.8 ; 7.6) | 3.8 (1.3 ; 6.2) | 0.002 |
| 1 - 5 | 2.8 (1.4 ; 5.7) | 4.2 (2.8 ; 6.2) | 1.4 (-1.2 ; 4.0) | 0.304 |
| at least 6 | 1.2 (0.6 ; 2.6) | 5.4 (3.2 ; 9.0) | 4.1 (1.2 ; 7.1) | 0.006 |
| Body mass index (kg/m2) |  |  |  |  |
| < 25 | 1.9 (0.9 ; 3.9) | 4.0 (2.8 ; 5.7) | 2.1 (0.2 ; 4.1) | 0.033 |
| ≥ 25 | 2.1 (1.1 ; 3.8) | 6.9 (4.3 ; 10.9) | 4.8 (1.4 ; 8.3) | 0.006 |
| Waist-to-height (WHtR) ratio |  |  |  |  |
| < 0.5 | 2.1 (1.1 ; 4.3) | 4.1 (2.8 ; 6.0) | 1.9 (-0.2 ; 4.1) | 0.081 |
| ≥ 0.5 - 0.6 | 1.1 (0.5 ; 2.4) | 5.1 (3.4 ; 7.5) | 4.0 (1.8 ; 6.2) | < 0.001 |
| ≥ 0.6 | 2.6 (1.0 ; 6.7) | 8.8 (3.7 ; 19.7) | 6.3 (-1.6 ; 14.2) | 0.119 |
|  |  |  |  |  |
